# Supplementary material for: The C9orf72 protein interacts with Rab1a and the ULK1 complex to regulate initiation of autophagy
Source: EMBO J. 2016 Jun 22;35(15):1656–76. doi: 10.15252/embj.201694401 (PMC4969571; doi:10.15252/embj.201694401)
Supplement: Supplementary file 1 — Appendix [file EMBJ-35-1656-s001.pdf]

The C9orf72 protein interacts with Rab1a and the ULK1 complex to regulate initiation of autophagy

Authors: Christopher P. Webster[1,4], Emma F. Smith[1,4], Claudia S. Bauer[1], Annekathrin Moller[1], Guillaume M. Hautbergue[1], Laura Ferraiuolo[1], Monika Myszczynska[1], Adrian Higginbottom[1], Matthew J. Walsh[1], Alexander J. Whitworth[2,5], Brian K. Kaspar[3], Kathrin Meyer[3], Pamela J. Shaw[1], Andrew J. Grierson[1,6], and Kurt J. De Vos[1,6,7]

Appendix Supplementary information:

Appendix Figure S1: Single C9orf72 siRNAs impair initiation of autophagy

Appendix Figure S2: Quantification of siRNA knock down.

Appendix Figure S3: Identification of input in GST pull down experiments by mass spectrometry.

Appendix Figure S4: C9orf72 mRNA levels in iNeurons.

Appendix Table S1. Patient information.

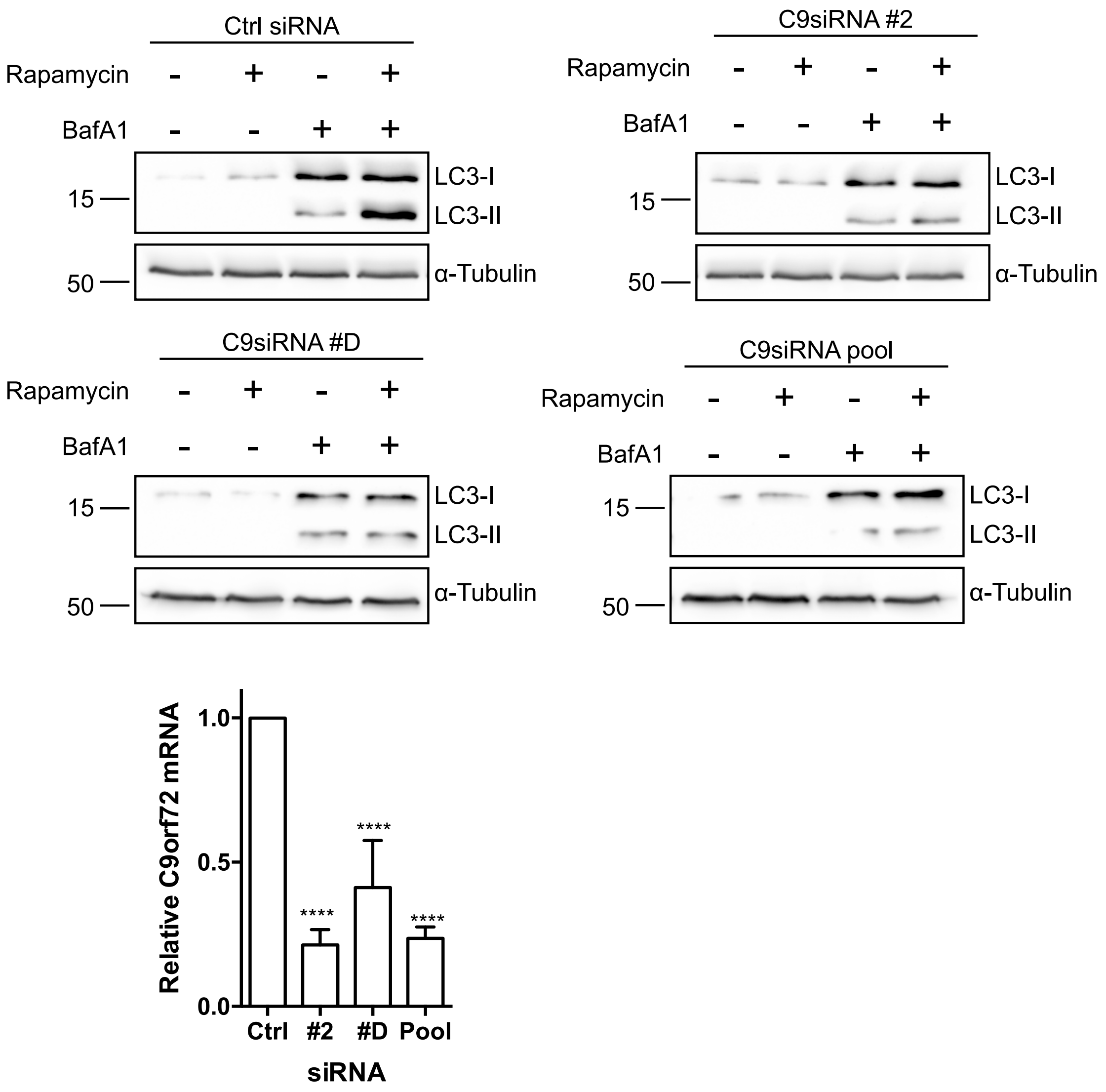

Appendix Figure S1: Single C9orf72 siRNAs impair initiation of autophagy.

HEK293 cells treated with non-targeting (Ctrl), C9orf72 siRNA #2, C9orf72 siRNA #D, or a pool of C9orf72 siRNA #2 and #D were incubated with vehicle, rapamycin, BafA1, or BafA1+Rapamycin and levels of LC3-I and II were determined on immunoblots. Both single C9orf72 siRNAs #2 and #D knocked down C9orf72 expression and inhibited initiation of autophagy similar to the pool of siRNAs. Knock down efficiency was tested by RT-QPCR from 3 independent experiments (including the experiment shown here). Statistical significance was determined by one-way ANOVA with Fisher's LSD test. *** $p \leq 0.001$, **** $p \leq 0.0001$

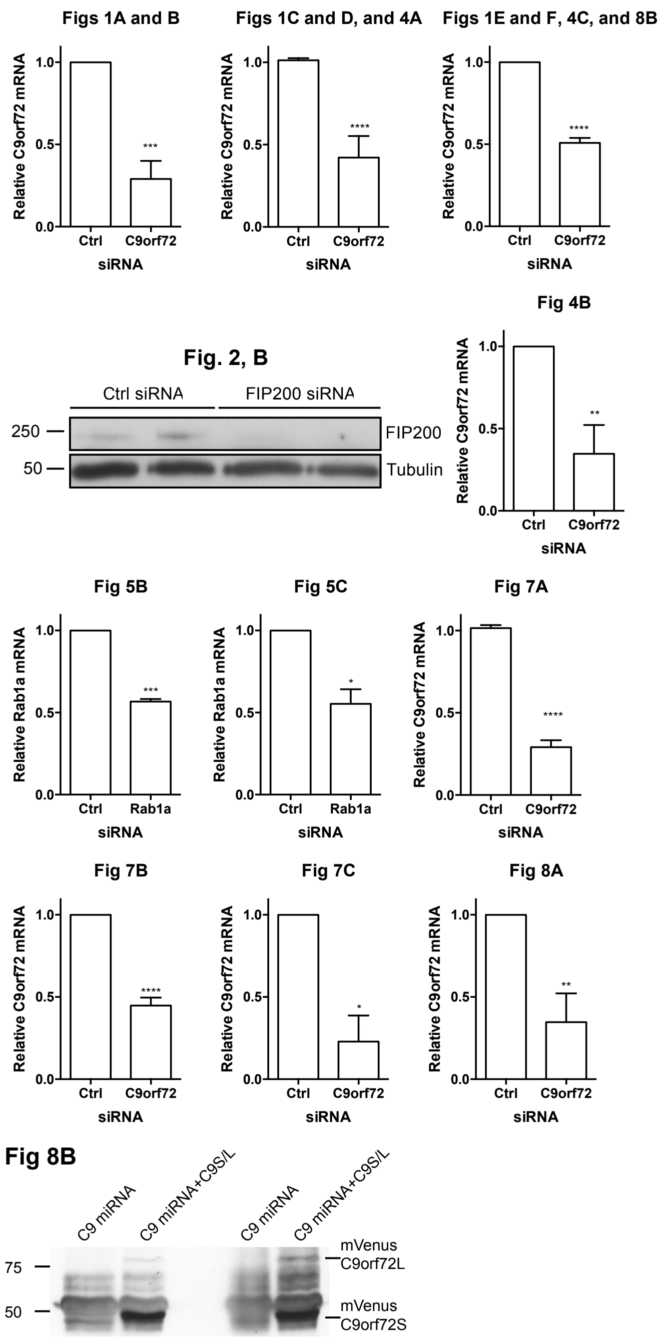

Appendix Figure S2: Q uantification of siRNA knock down. Knockdown of C9orf72 and Rab1a was quantified by RT-qPCR. FIP200 knock down and re-expression of human C9orf72 in neurons was determined by immunoblot. was Matching figures are indicated in the title of each panel. Statistical significance was determined by unpaired t-test. * $p \leq 0.05$, ** $p \leq 0.01$, *** $p \leq 0.001$, **** $p \leq 0.0001$.

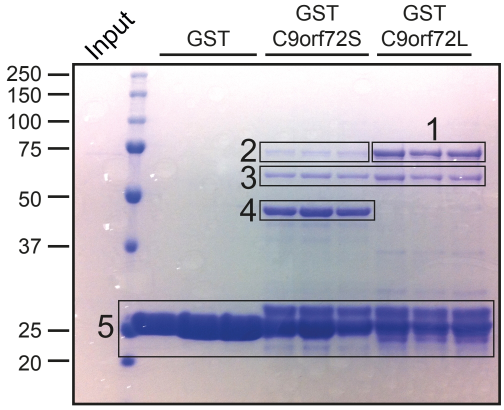

Band	Identity
1	GST-C9orf72L
2	DnaK Chaperonin E.coli
3	CH60 E.coli - 60 kDa Chaperonin
4	GST-C9orf72S
5	GST/30S ribosome Ecoli

Appendix Figure S3: Identification of input in GST pull down experiments by mass spectrometry. Indicated bands were excised from a Coomassie stained gel and proteins identified by LC-MS/MS.

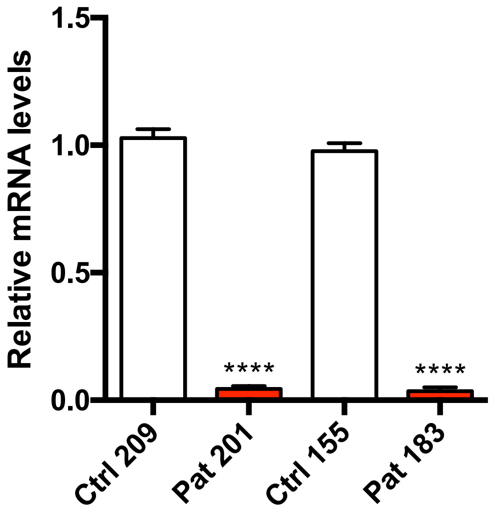

Appendix Figure S4: C9orf72 mRNA levels in iNeurons. The levels of C9orf72 mRNA were determined by RT-qPCR (Statistical significance was determined by one-way ANOVA with Fisher's LSD test. (**** $p \leq 0.0001$; $n = 4$)

Appendix Table S1. Patient information

Sample ID	Ethnicity	Gender	Mutation	Age at diagnosis and biopsy collection (y)
**155**	Caucasian	Male	Non-ALS	40
**183**	Caucasian	Male	C9orf72	49
**209**	Caucasian	Female	Non-ALS	69
**201**	Caucasian	Female	C9orf72	66
