# Supplementary material for: The C9orf72 protein interacts with Rab1a and the ULK1 complex to regulate initiation of autophagy
Source: EMBO J. 2016 Jun 22;35(15):1656–76. doi: 10.15252/embj.201694401 (PMC4969571; doi:10.15252/embj.201694401)
Supplement: Supplementary file 2 — Expanded View Figures PDF [file EMBJ-35-1656-s002.pdf]

Expanded View Figures

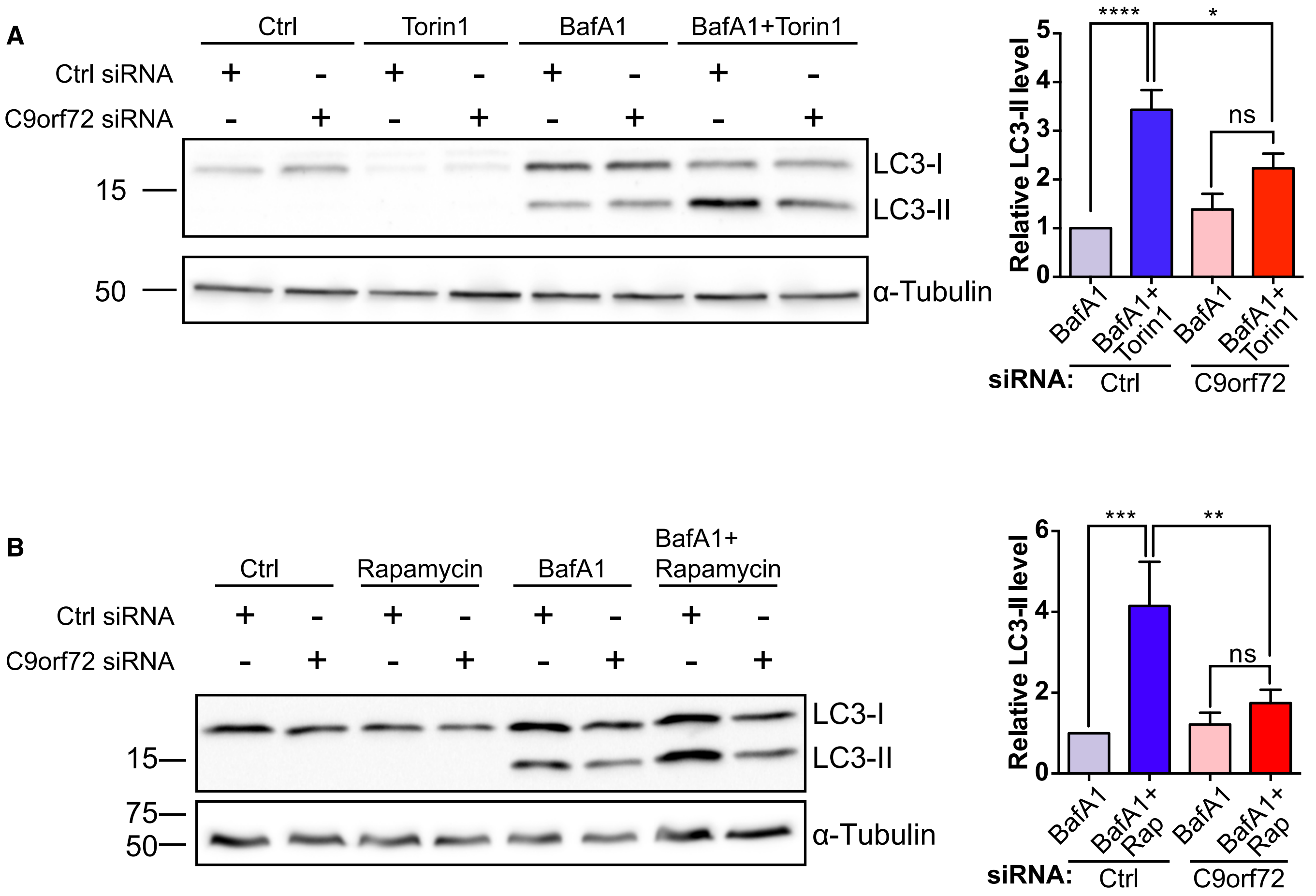

Figure EV1. C9orf72 regulates the initiation of autophagy.

A HEK293 cells treated with non-targeting (Ctrl) or C9orf72 siRNA were incubated with vehicle (Ctrl), Torin1, BafA1, or BafA1 + Torin1 for 3 h, and levels of LC3-I and II were determined on immunoblots. Levels of LC3-II were normalized against α-tubulin and are shown relative to the BafA1-treated sample (mean ± SEM; one-way ANOVA with Fisher's LSD test: ns, not significant; $^*P \leq 0.05$, $^{****}P \leq 0.0001$; $N = 3$ experiments). These data are also shown in Fig 1C.

B HEK293 cells treated with non-targeting (Ctrl) or C9orf72 siRNA were incubated with vehicle (Ctrl), rapamycin, BafA1, or BafA1 + rapamycin for 6 h, and levels of LC3-I and II were determined on immunoblots. Levels of LC3-II were normalized against α-tubulin and are shown relative to the BafA1-treated sample (mean ± SEM; one-way ANOVA with Fisher's LSD test: ns, not significant; $^{**}P \leq 0.01$, $^{***}P \leq 0.001$; $N = 3$ experiments). These data are also shown in Fig 1D.

Figure EV2. C9orf72 interacts with the ULK1 initiation complex.

A HeLa cells were transfected with FLAG-FIP200, Myc-C9orf72S and Myc-C9orf72L or co-transfected with FLAG-FIP200 and either Myc-C9orf72S or Myc-C9orf72L as indicated. Transfections were laced with mVenus to enable identification of transfected cells for analysis (green). Transfected cells were probed with both anti-FLAG and anti-Myc antibodies and processed for PLA. PLA proximity signals per cell (red) were determined (mean ± SEM; one-way ANOVA with Fisher's LSD test: $^{****}P \leq 0.0001$; N (cells) = FLAG-FIP200: 18, Myc-C9orf72S: 22, Myc-C9orf72L: 18, FLAG-FIP200 + Myc-C9orf72S: 18, FLAG-FIP200 + Myc-C9orf72L: 17). Scale bar = 20 μm.

B HeLa cells were transfected with HA-ULK1, Myc-C9orf72S and Myc-C9orf72L or co-transfected with HA-ULK1 and either Myc-C9orf72S or Myc-C9orf72L as indicated. Transfections were laced with mVenus to enable identification of transfected cells for analysis (green). Transfected cells were probed with both anti-HA and anti-myc antibodies and processed for PLA. PLA proximity signals per cell (red) were determined (mean ± SEM; one-way ANOVA with Fisher's LSD test: $^{****}P \leq 0.0001$; N (cells) = HA-ULK1: 20, Myc-C9orf72S: 21, Myc-C9orf72L: 20, HA-ULK1 + Myc-C9orf72S: 22, HA-ULK1 + Myc-C9orf72L: 20). Scale bar = 20 μm.

C HeLa cells were transfected with Myc-ATG13, EGFP-C9orf72S and EGFP-C9orf72L or co-transfected with Myc-ATG13 and either EGFP-C9orf72S or EGFP-C9orf72L. The ATG13 transfection was laced with mVenus to enable detection of transfected cells for analysis (green). Transfected cells were probed with both anti-EGFP and anti-myc antibodies and processed for PLA. PLA proximity signals per cell (red) were determined (mean ± SEM; one-way ANOVA with Fisher's LSD test: $^{****}P \leq 0.0001$; N (cells) = ATG13: 11, EGFPc2-C9orf72S: 11, EGFPc2-C9orf72L: 10, Myc-ATG13 + EGFPc2-C9orf72S: 11, Myc-ATG13 + EGFPc2-C9orf72L: 11). Scale bar = 20 μm.

 © 2016 The Authors

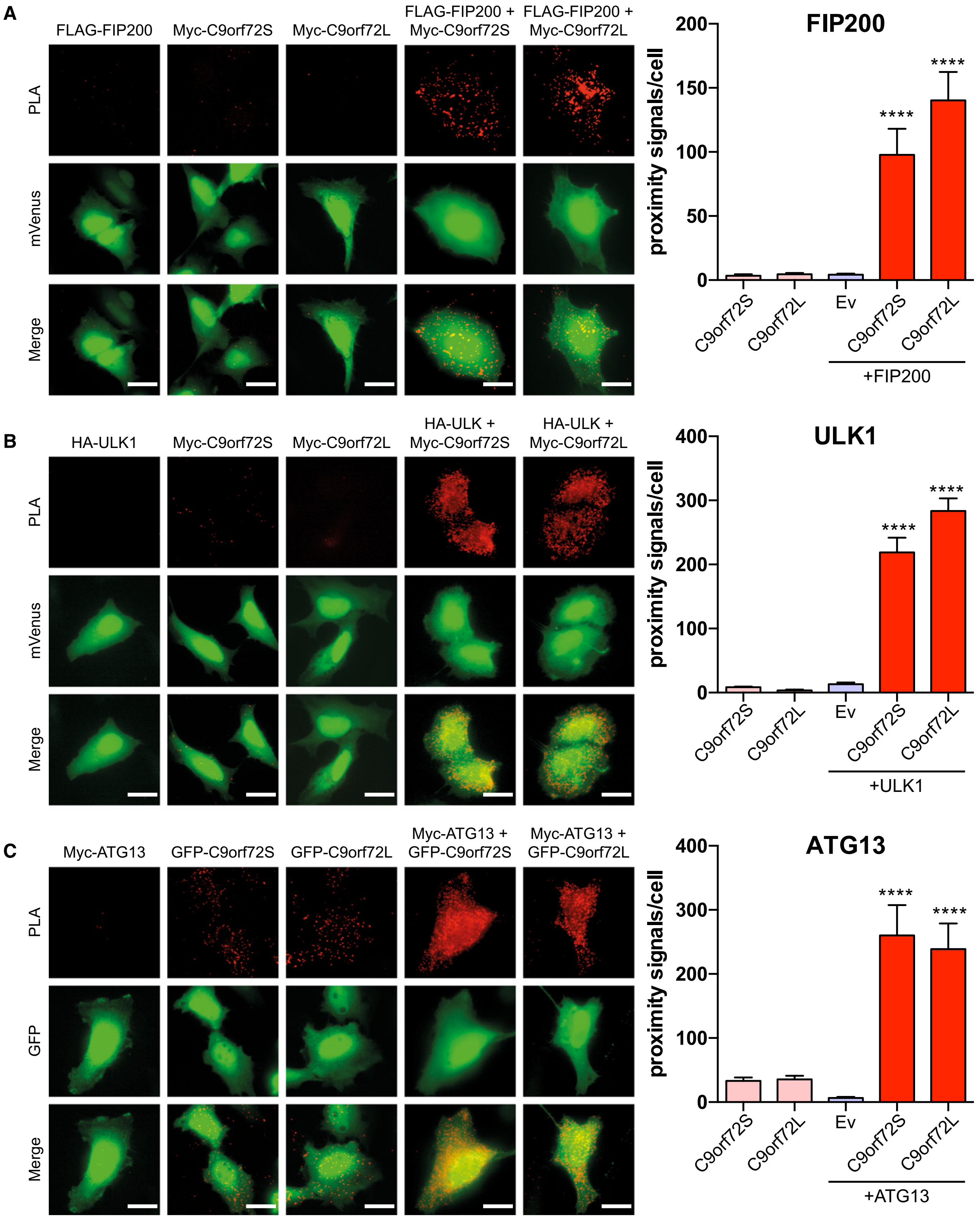

A
FLAG-FIP200
Myc-C9orf72S
Myc-C9orf72L
FLAG-FIP200 + Myc-C9orf72S
FLAG-FIP200 + Myc-C9orf72L
PLA
mVenus
Merge
FIP200
proximity signals/cell
200
150
100
50
0
****
****
C9orf72S
C9orf72L
Ev
C9orf72S
C9orf72L
+FIP200
B
HA-ULK1
Myc-C9orf72S
Myc-C9orf72L
HA-ULK + Myc-C9orf72S
HA-ULK + Myc-C9orf72L
PLA
mVenus
Merge
ULK1
proximity signals/cell
400
300
200
100
0
****
****
C9orf72S
C9orf72L
Ev
C9orf72S
C9orf72L
+ULK1
C
Myc-ATG13
GFP-C9orf72S
GFP-C9orf72L
Myc-ATG13 + GFP-C9orf72S
Myc-ATG13 + GFP-C9orf72L
PLA
GFP
Merge
ATG13
proximity signals/cell
400
300
200
100
0
****
****
C9orf72S
C9orf72L
Ev
C9orf72S
C9orf72L
+ATG13

Figure EV2.

Figure EV3. C9orf72 regulates translocation of the ULK1 complex to the phagophore via Rab1a.

A HeLa cells were co-transfected with empty vector (EV), FLAG-C9orf72S or FLAG-C9orf72S (green), Myc-Rab1aWT or dominant negative Myc-Rab1aS25N (DN), and mCherry-FIP200 (red). 24 h post-transfection, cells were treated with Torin1 (250 nM; 3 h). Translocation of the ULK1 complex was quantified as the number of mCherry-FIP200-positive puncta per cell from 7 independent experiments (mean ± SEM; one-way ANOVA with Fisher's LSD test: ns, not significant, $^{**}P \leq 0.01$, $^{****}P \leq 0.0001$; *N* (cells) = WT/EV: 142; WT/EV/Torin1: 73; WT/C9orf72L: 108; WT/C9orf72S: 123; DN/EV: 159; DN/EV/Torin1: 150; DN/C9orf72L: 159; DN/C9orf72S: 123). Scale bar = 10 μm.

B HeLa cells were co-transfected with empty vector (EV), FLAG-C9orf72S or FLAG-C9orf72S (red), Myc-Rab1aWT or dominant negative Myc-Rab1aS25N (DN), and EGFP-LC3 (green). 24 h post-transfection, cells were treated with Torin1 (250 nM; 3 h). Autophagosomes were quantified as the number of EGFP-LC3-positive puncta per cell from 2 independent experiments (mean ± SEM; one-way ANOVA with Fisher's LSD test: ns, not significant, $^{**}P \leq 0.01$, $^{****}P \leq 0.0001$; *N* (cells) = WT/EV: 51; WT/EV/Torin1: 53; WT/C9orf72L: 56; WT/C9orf72S: 41; DN/EV: 53; DN/EV/Torin1: 53; DN/C9orf72L: 55; DN/C9orf72S: 42). Scale bar = 10 μm.

© 2016 The Authors

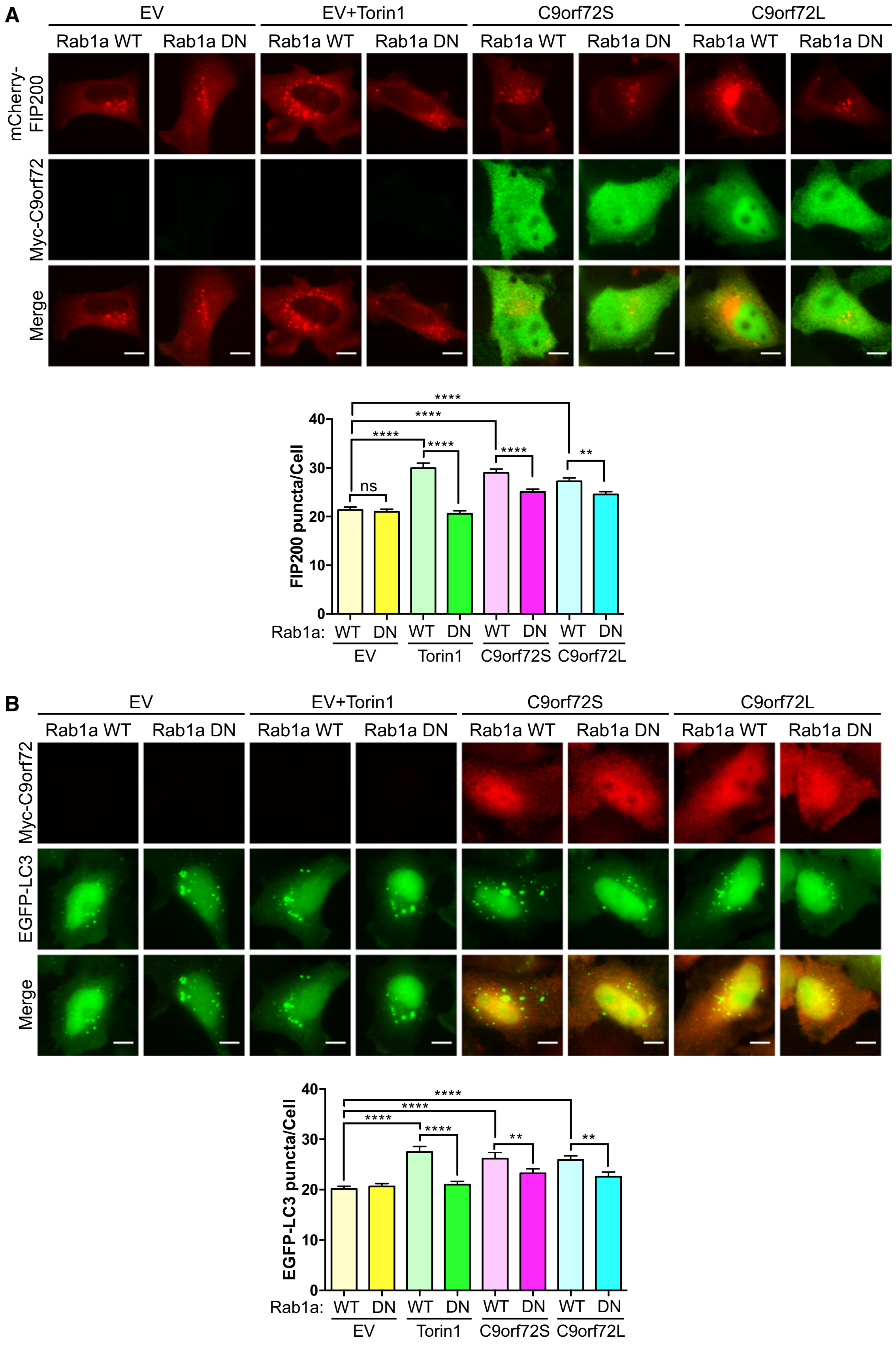
A
EV
EV+Torin1
C9orf72S
C9orf72L
Rab1a WT
Rab1a DN
mCherry-FIP200
Myc-C9orf72
Merge
FIP200 puncta/Cell
ns
****
**
Rab1a: WT DN WT DN WT DN WT DN
EV
Torin1
C9orf72S
C9orf72L
B
EV
EV+Torin1
C9orf72S
C9orf72L
Rab1a WT
Rab1a DN
Myc-C9orf72
EGFP-LC3
Merge
EGFP-LC3 puncta/Cell
****
**
Rab1a: WT DN WT DN WT DN WT DN
EV
Torin1
C9orf72S
C9orf72L

Figure EV3.
