# Supplementary figures and images for: The C9orf72 protein interacts with Rab1a and the ULK1 complex to regulate initiation of autophagy
Source: EMBO J. 2016 Jun 22;35(15):1656–76. doi: 10.15252/embj.201694401 (PMC4969571; doi:10.15252/embj.201694401)

Figure 1C

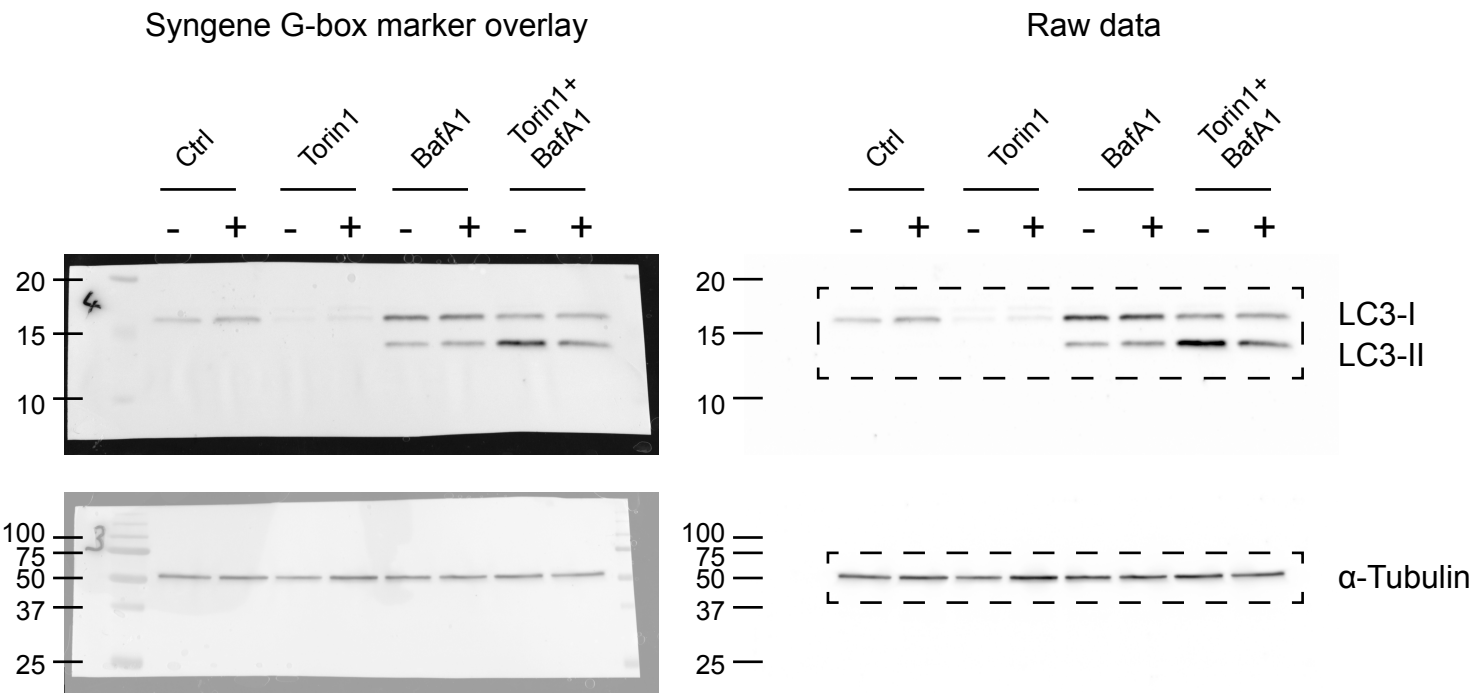

Figure 1D

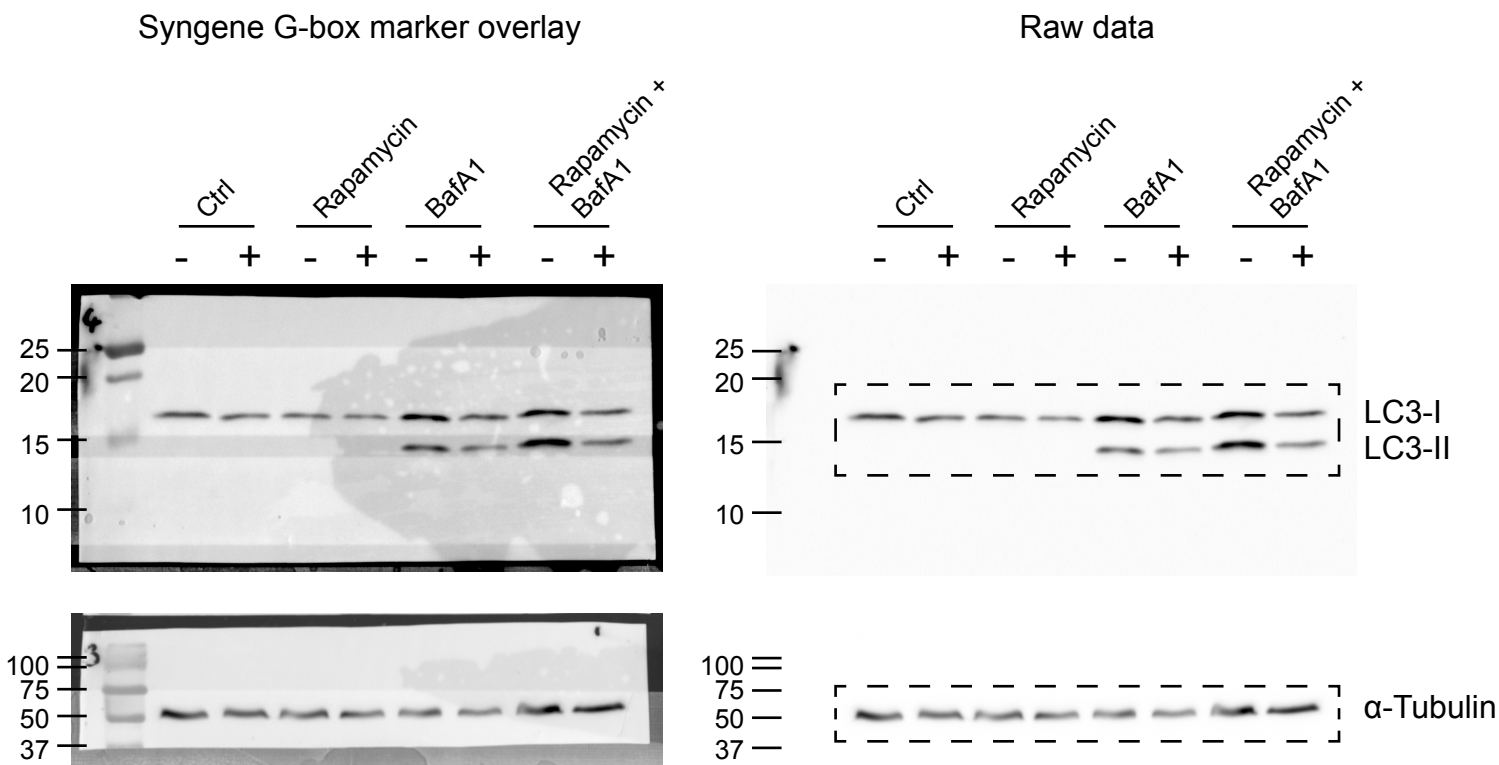

Supplement: Supplementary file 4 — Source Data for Figure 1 [file EMBJ-35-1656-s003.pdf]

Figure 2A

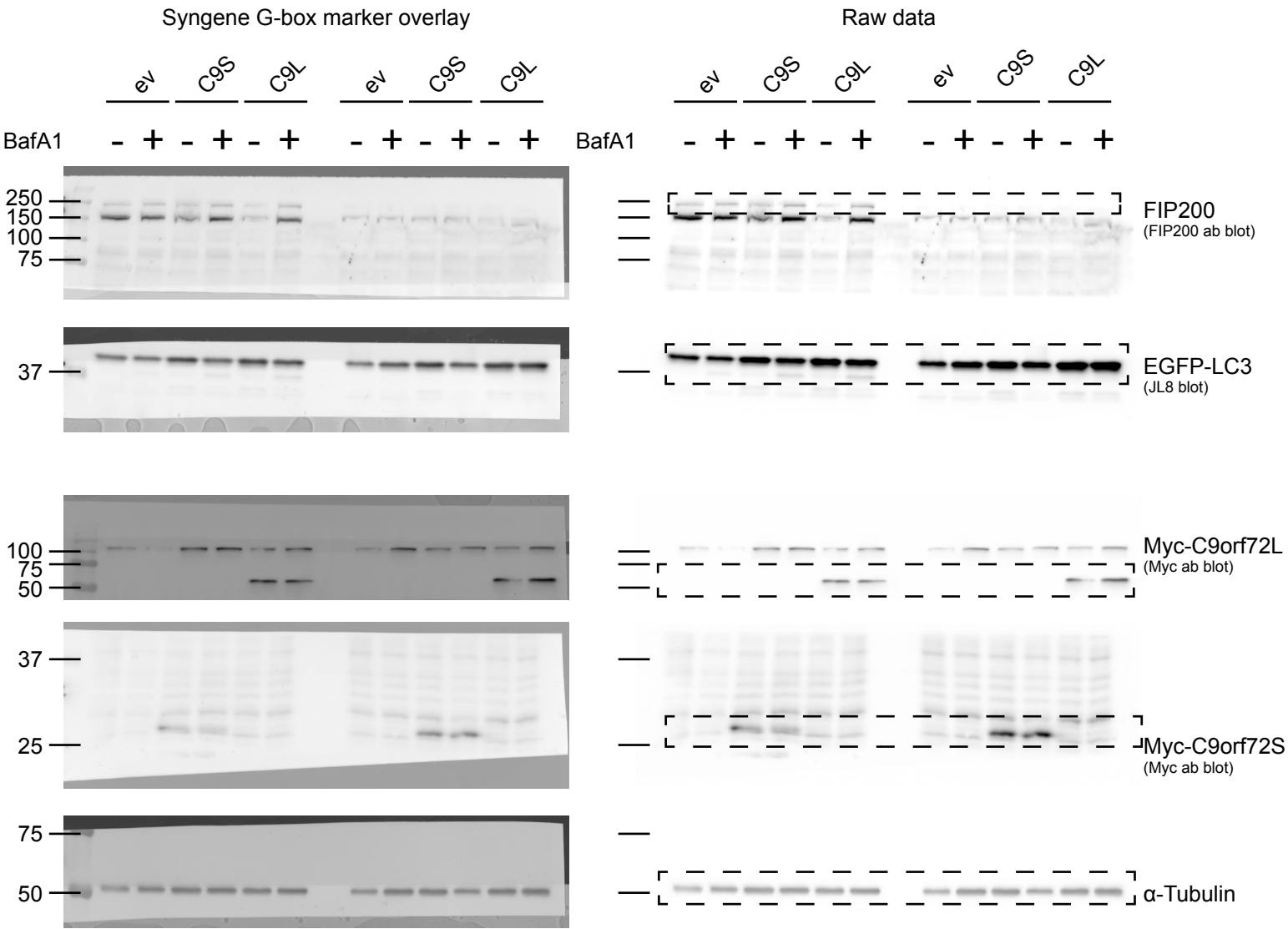

Supplement: Supplementary file 5 — Source Data for Figure 2 [file EMBJ-35-1656-s004.pdf]

Figure 3A

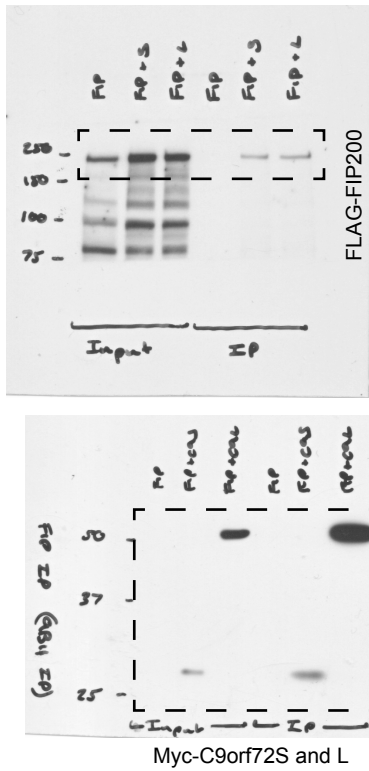

Figure 3B

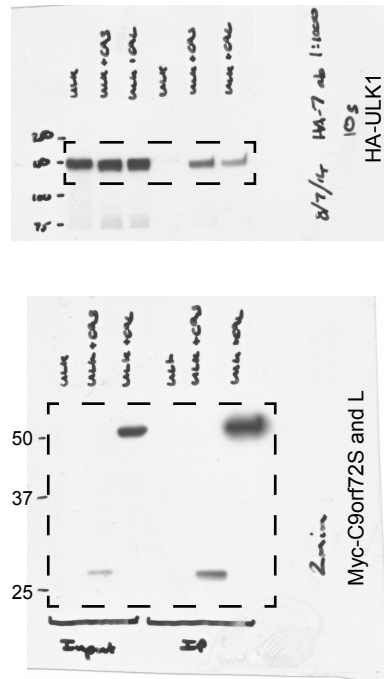

Figure 3C

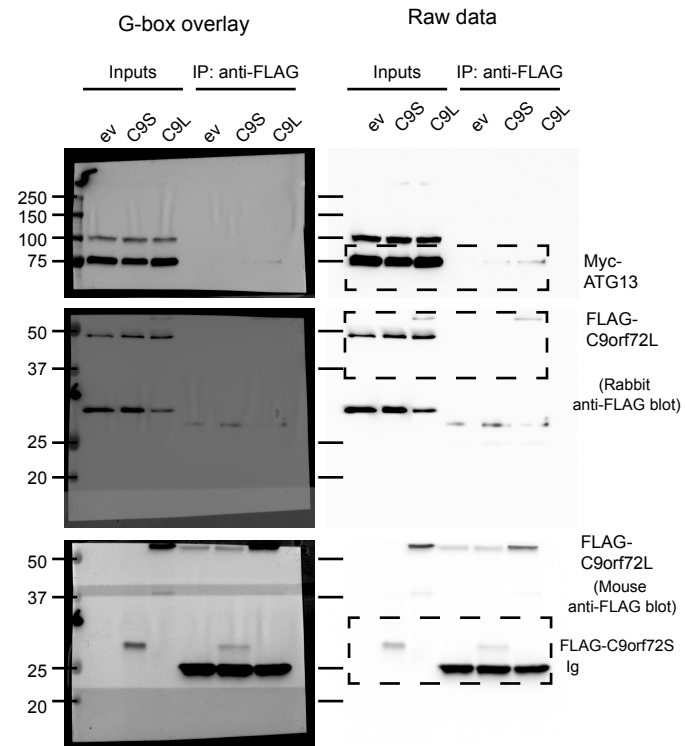

Figure 3D

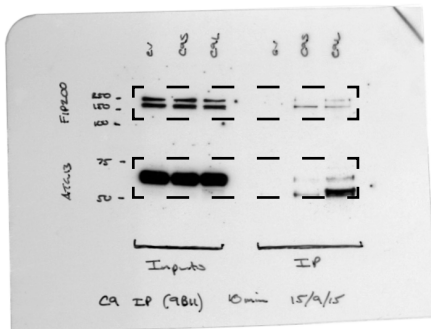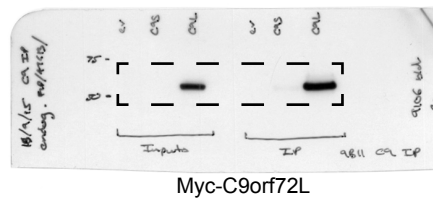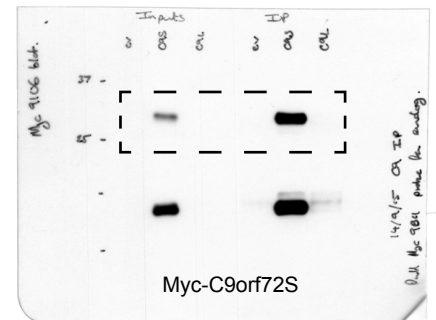

Figure 3E

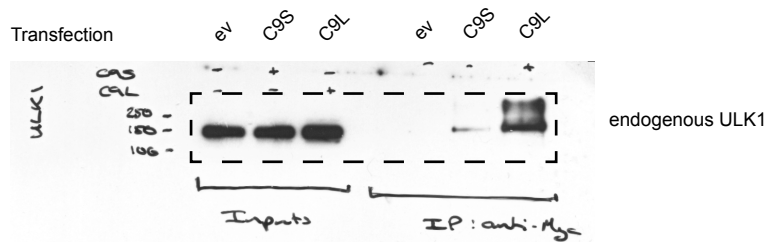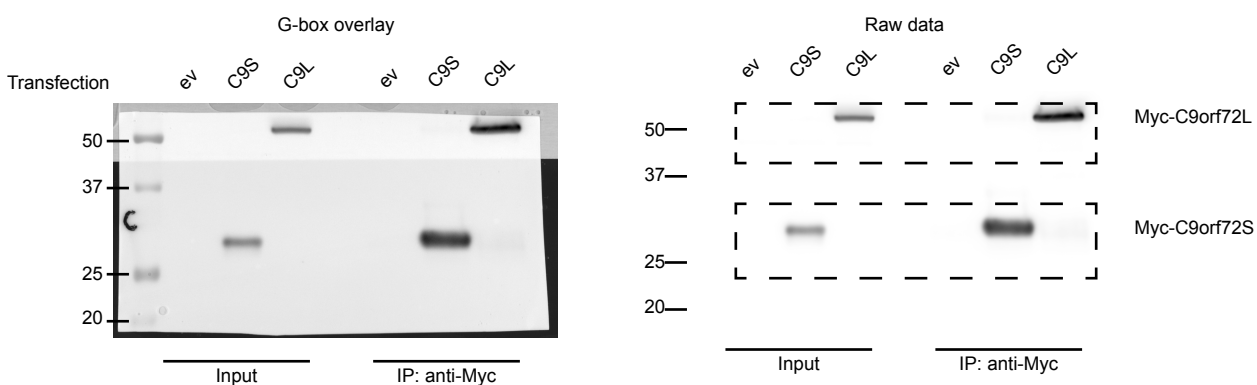

Supplement: Supplementary file 6 — Source Data for Figure 3 [file EMBJ-35-1656-s005.pdf]

Figure 4A

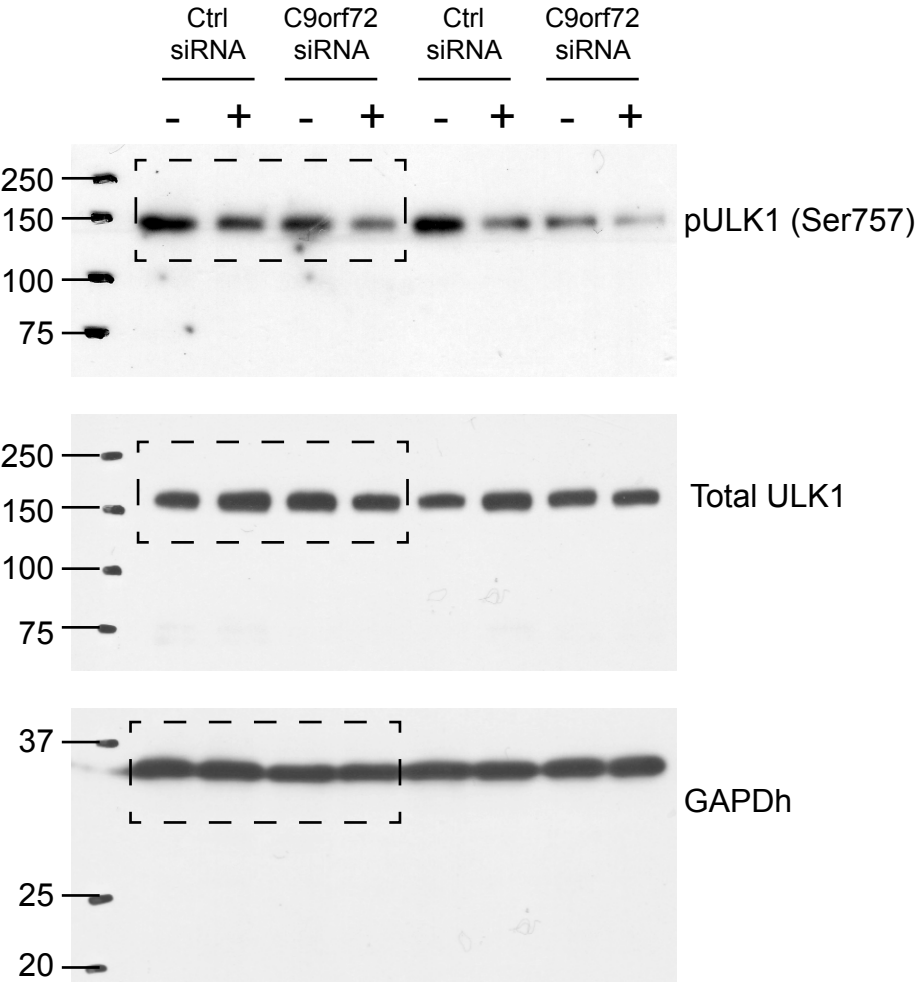

Supplement: Supplementary file 7 — Source Data for Figure 4 [file EMBJ-35-1656-s006.pdf]

Figure 6B

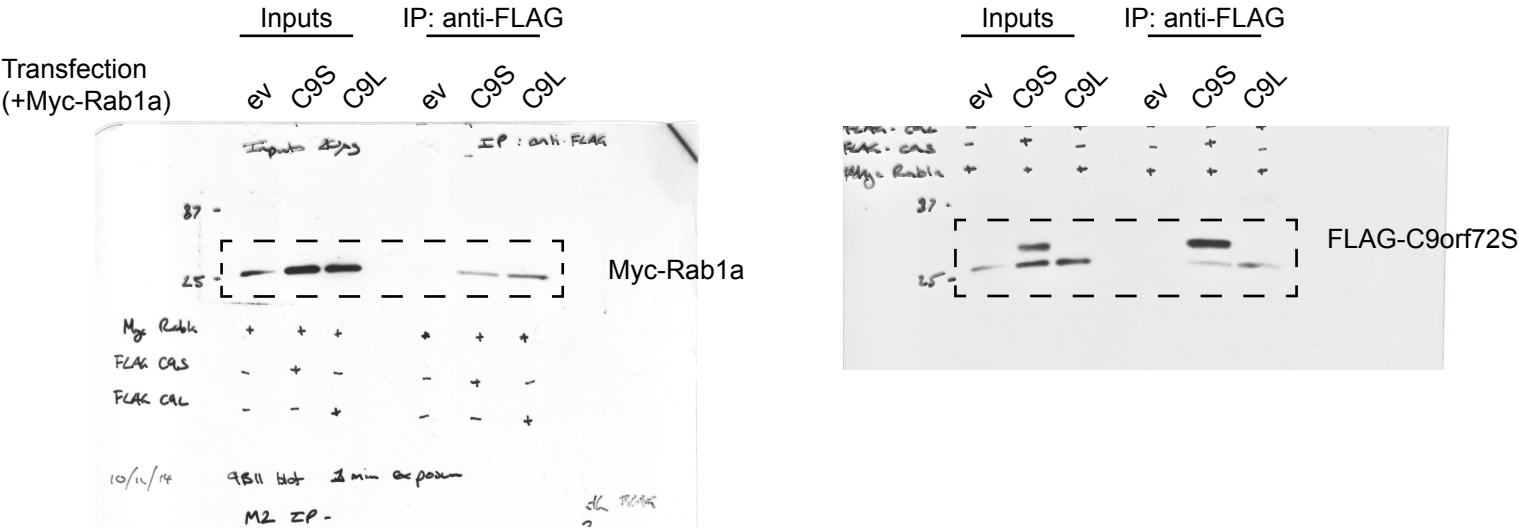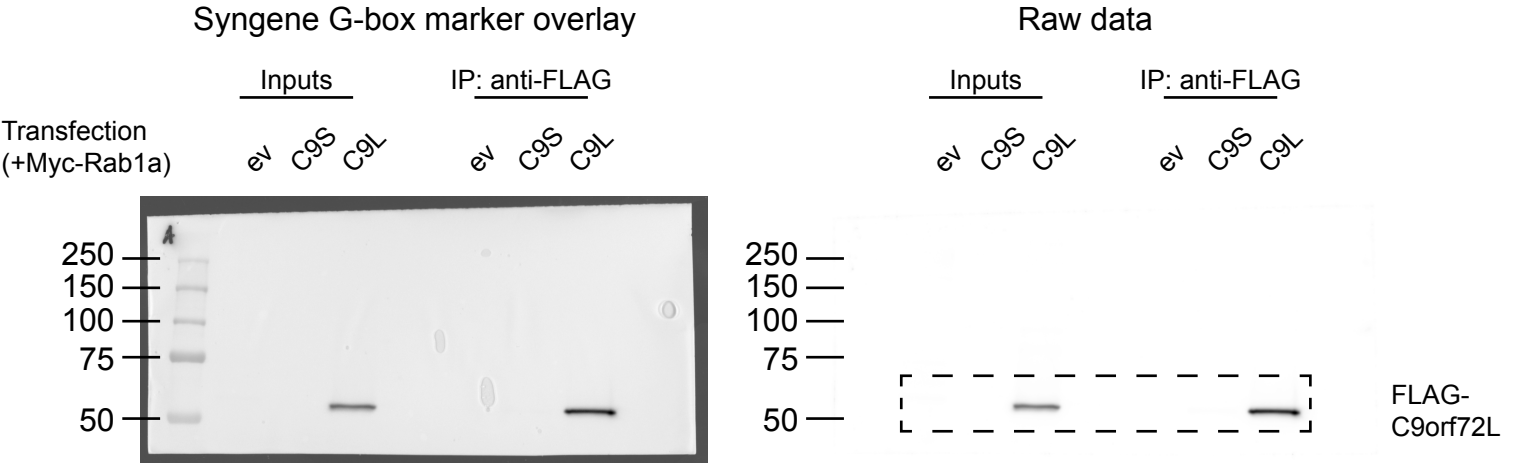

Supplement: Supplementary file 8 — Source Data for Figure 6 [file EMBJ-35-1656-s007.pdf]

Figure 9A

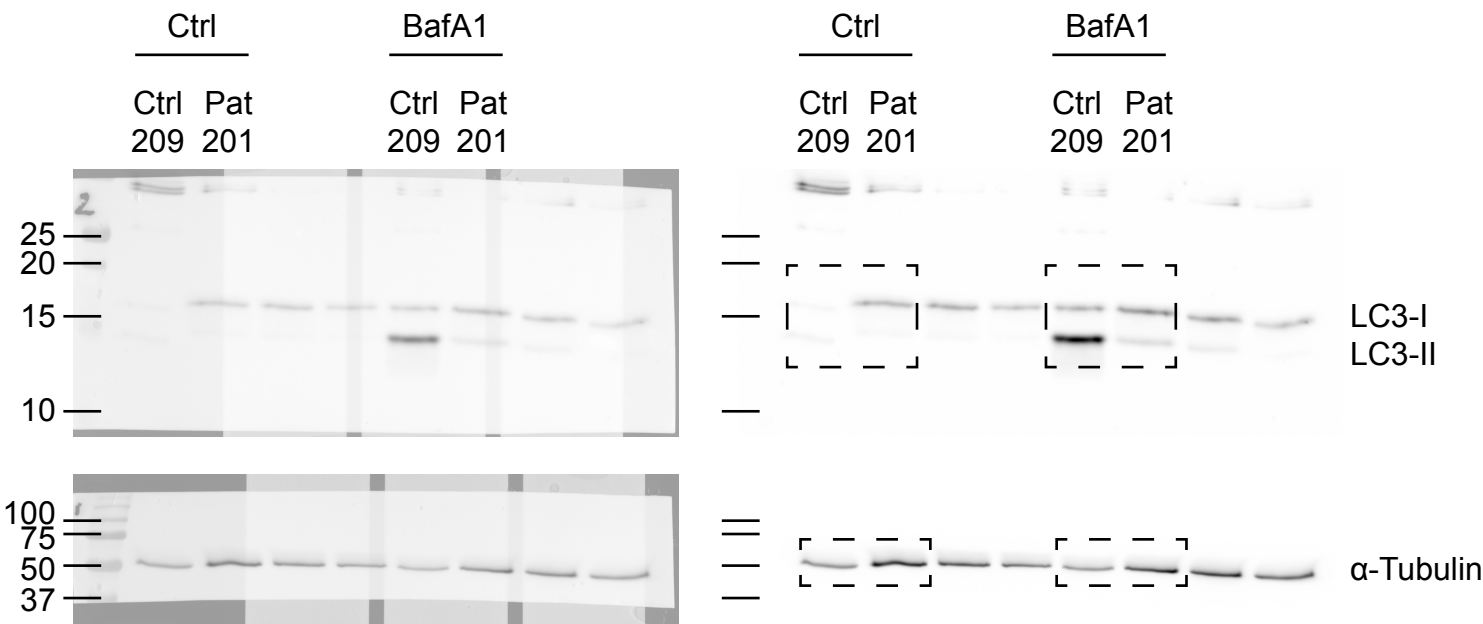

Figure 9B

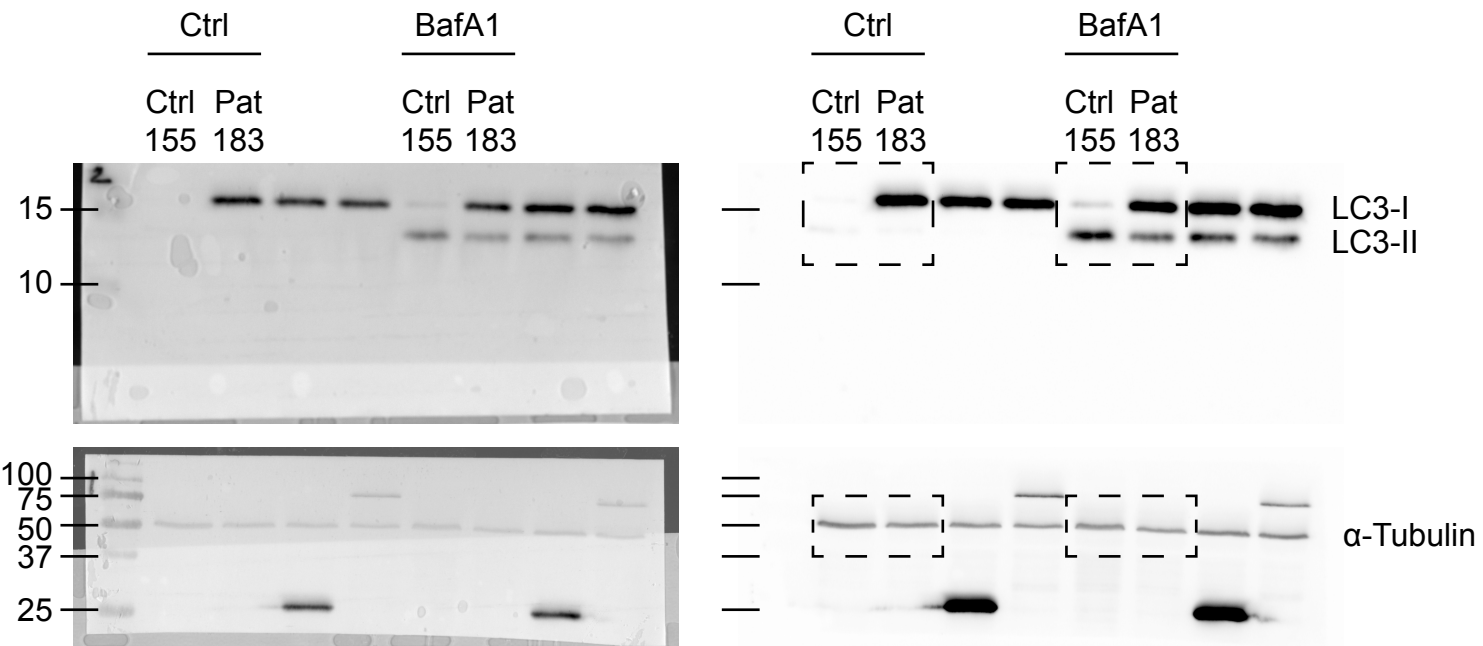

Supplement: Supplementary file 9 — Source Data for Figure 9 [file EMBJ-35-1656-s008.pdf]
